# Supplementary figures and images for: Improving germline transmission efficiency in chimeric chickens using a multi-stage injection approach
Source: PLoS One. 2021 Jun 4;16(6):e0247471. doi: 10.1371/journal.pone.0247471 (PMC8177527; doi:10.1371/journal.pone.0247471)

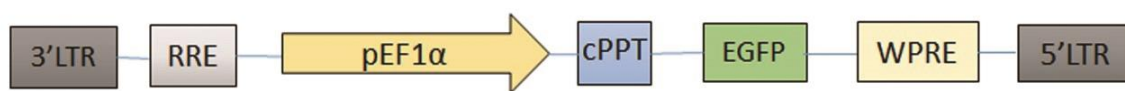

Supplement: S1 Fig — LTR, long terminal repeat; RRE, ref-responsive element; cPPT, central polypurine tract; pEF1a, human elongation factor-1 alpha promoter; EGFP, enhanced green fluorescent protein; WPRE, woodchuck hepatitis virus posttranscriptional control element. (PDF) [file pone.0247471.s001.pdf]

**Fig 2a**

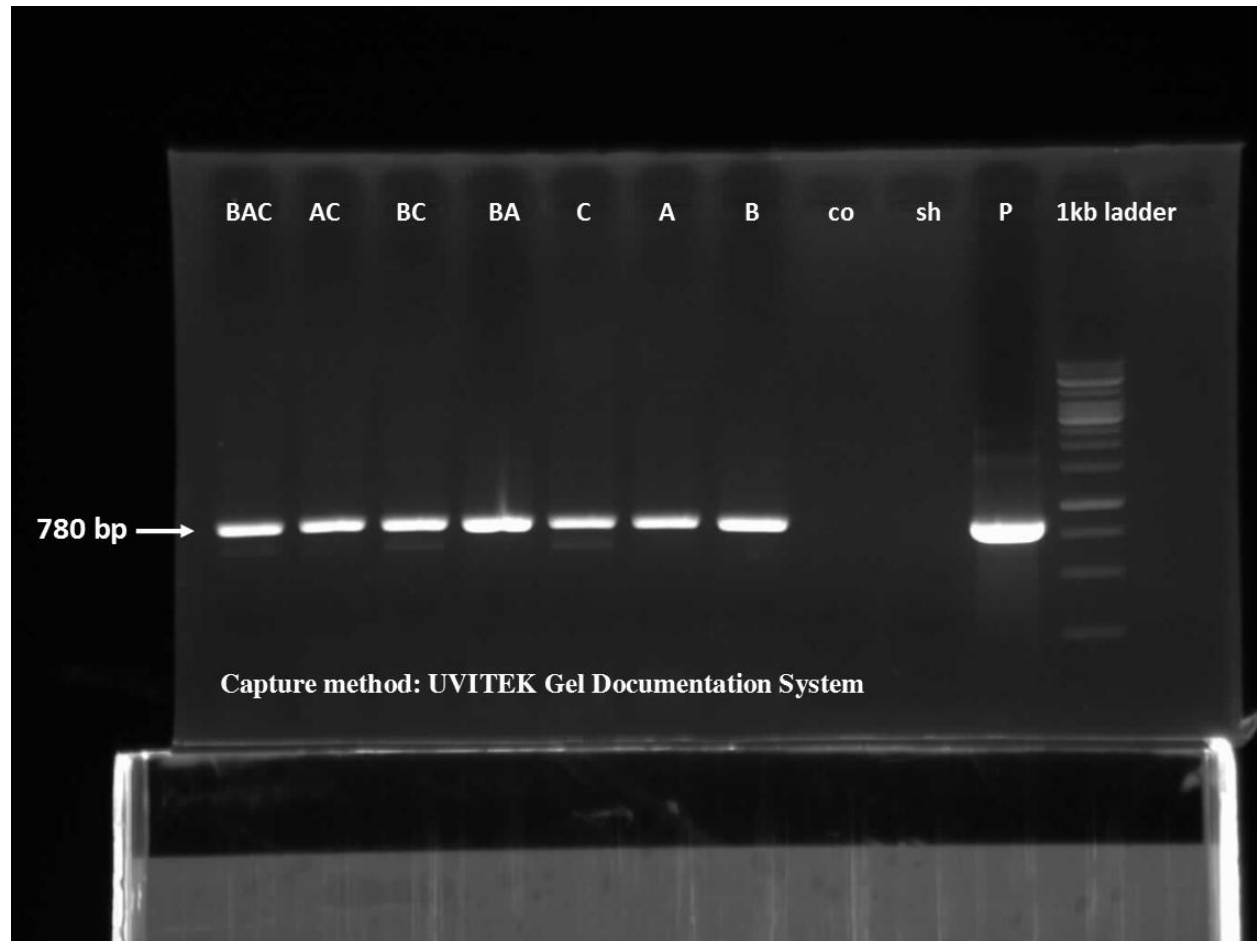

**Fig 4a ( $\beta$ -actin)**

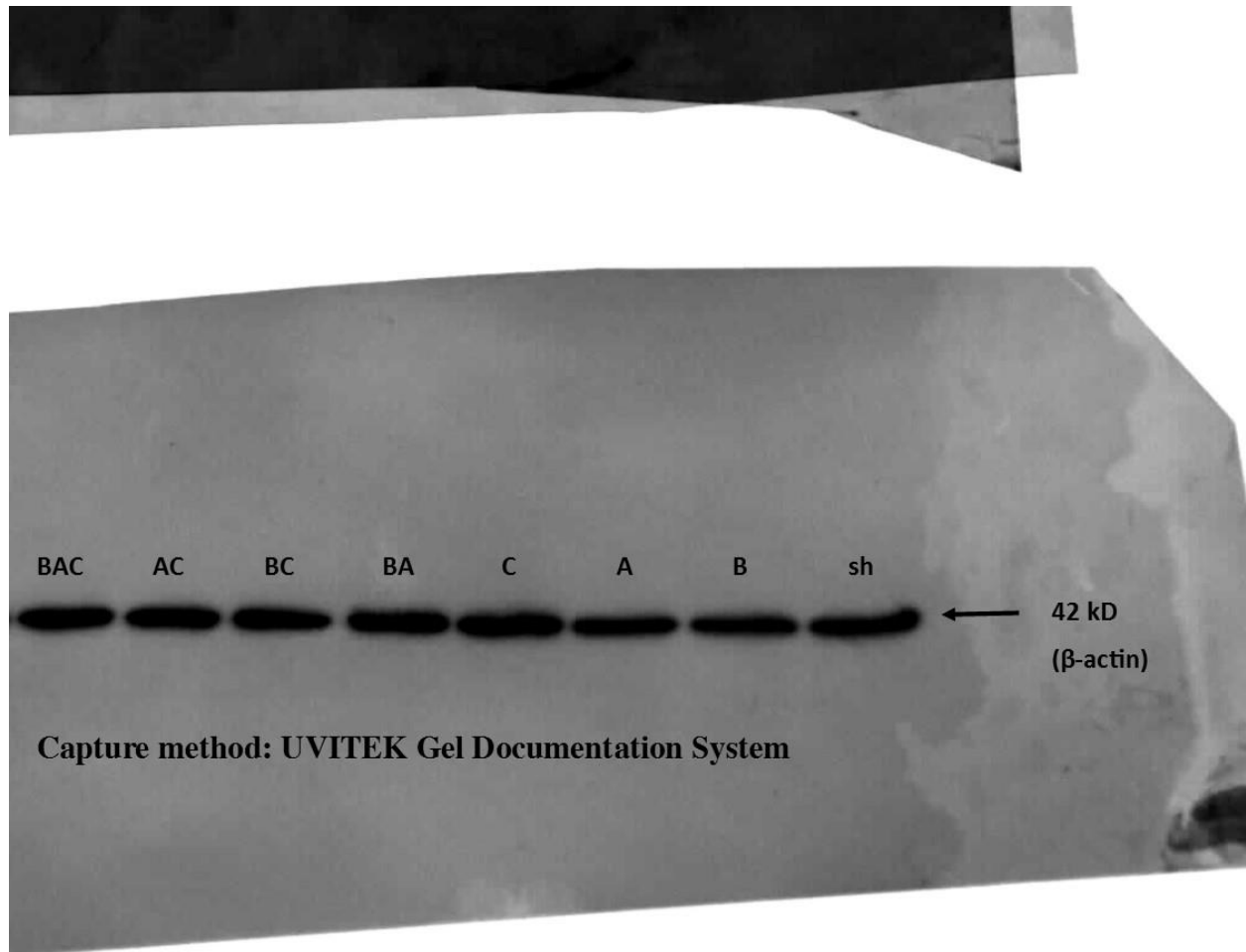

**Fig 4a (EGFP)**

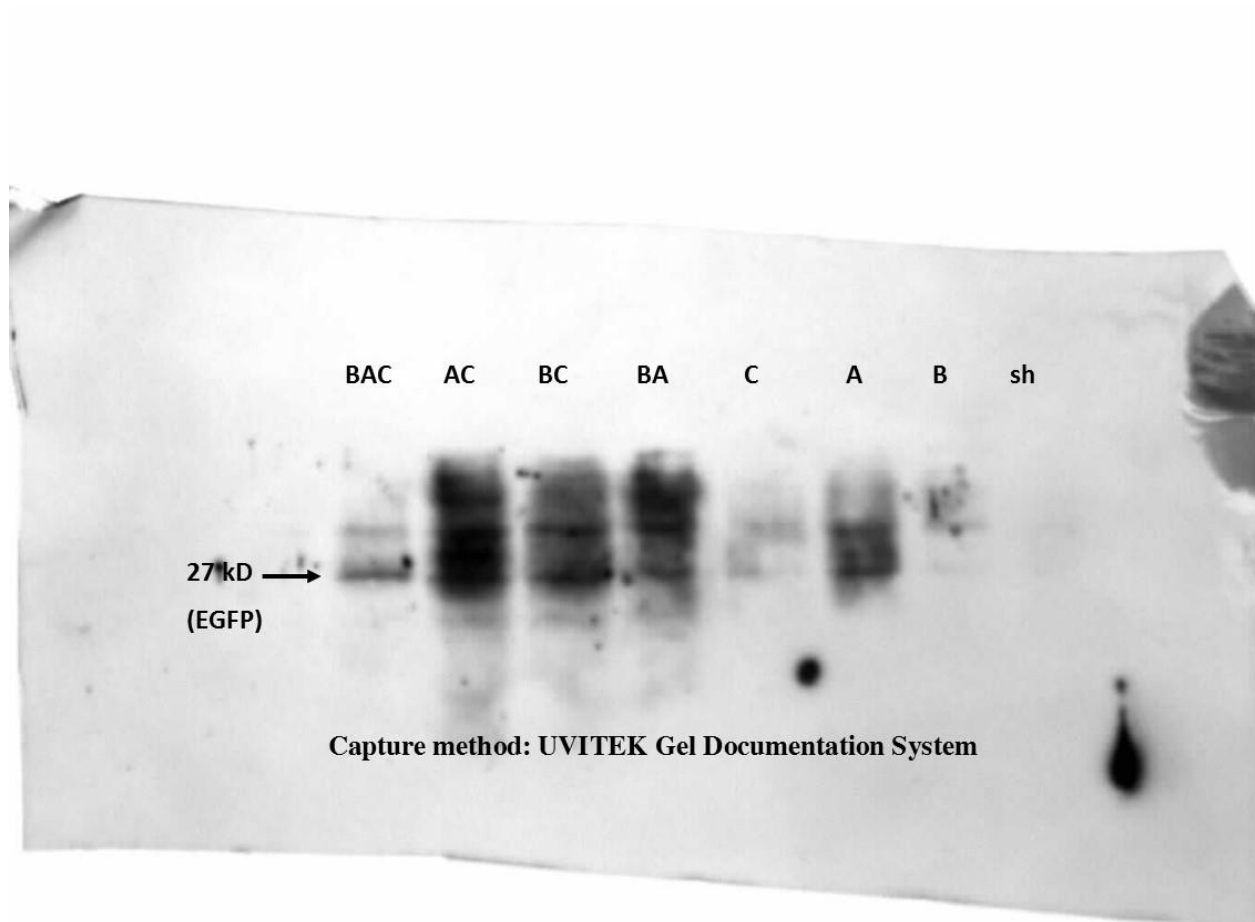

**Fig 5a**

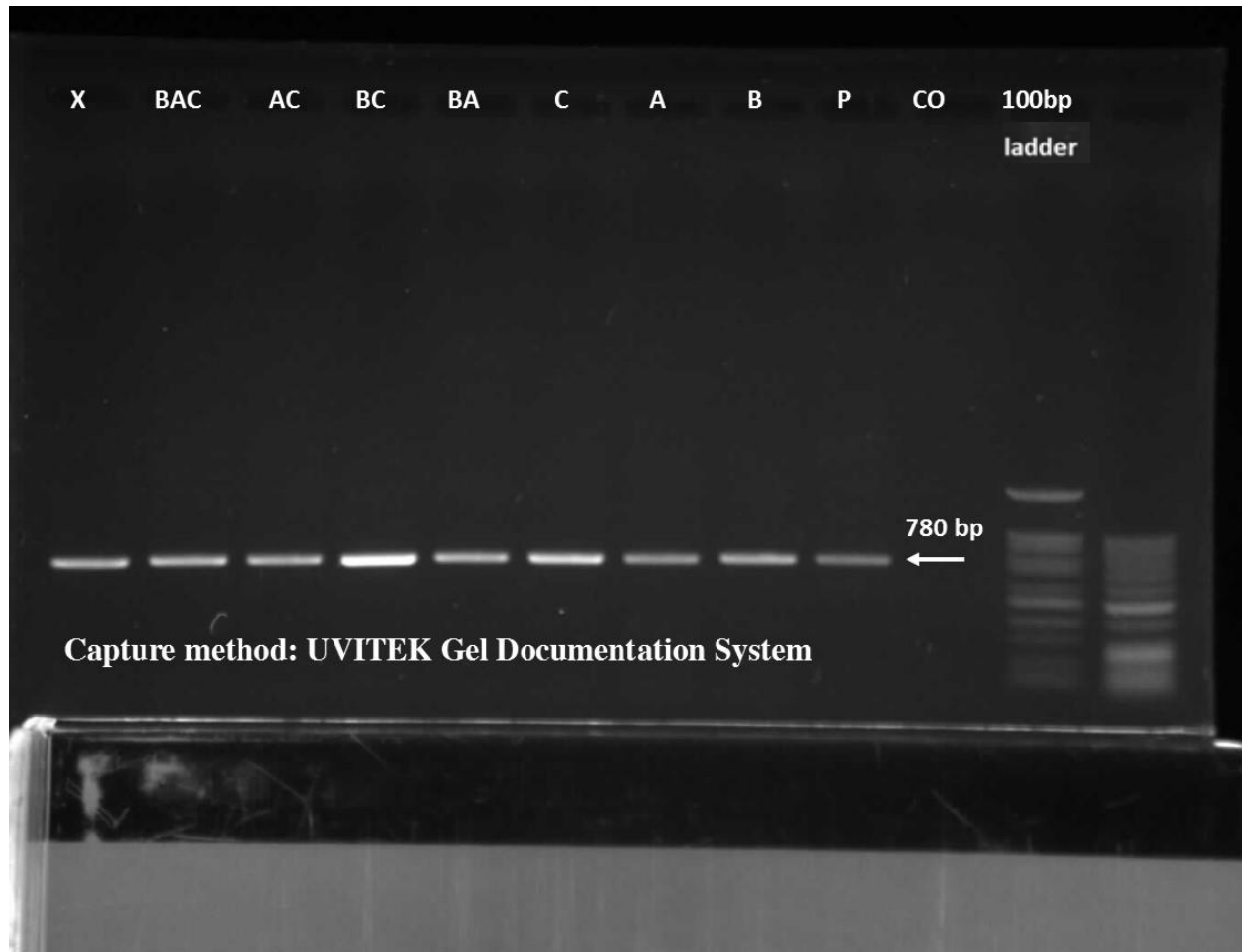

Supplement: S1 Raw images — (PDF) [file pone.0247471.s003.pdf]
