# Supplementary material for: Improving germline transmission efficiency in chimeric chickens using a multi-stage injection approach
Source: PLoS One. 2021 Jun 4;16(6):e0247471. doi: 10.1371/journal.pone.0247471 (PMC8177527; doi:10.1371/journal.pone.0247471)
Supplement: S2 Fig — Here, the sequence of genomic PCR product is compared with the EGFP sequence in pWPXL viral vector using SnapGene software. The result of sequence analysis confirmed the integration of the vector into the chicken genome. Upper sequence: EGFP ORF amplified from the genome-integrated pWPXL. Lower sequence: EGFP ORF in pWPXL viral vector. (PDF) [file pone.0247471.s002.pdf]

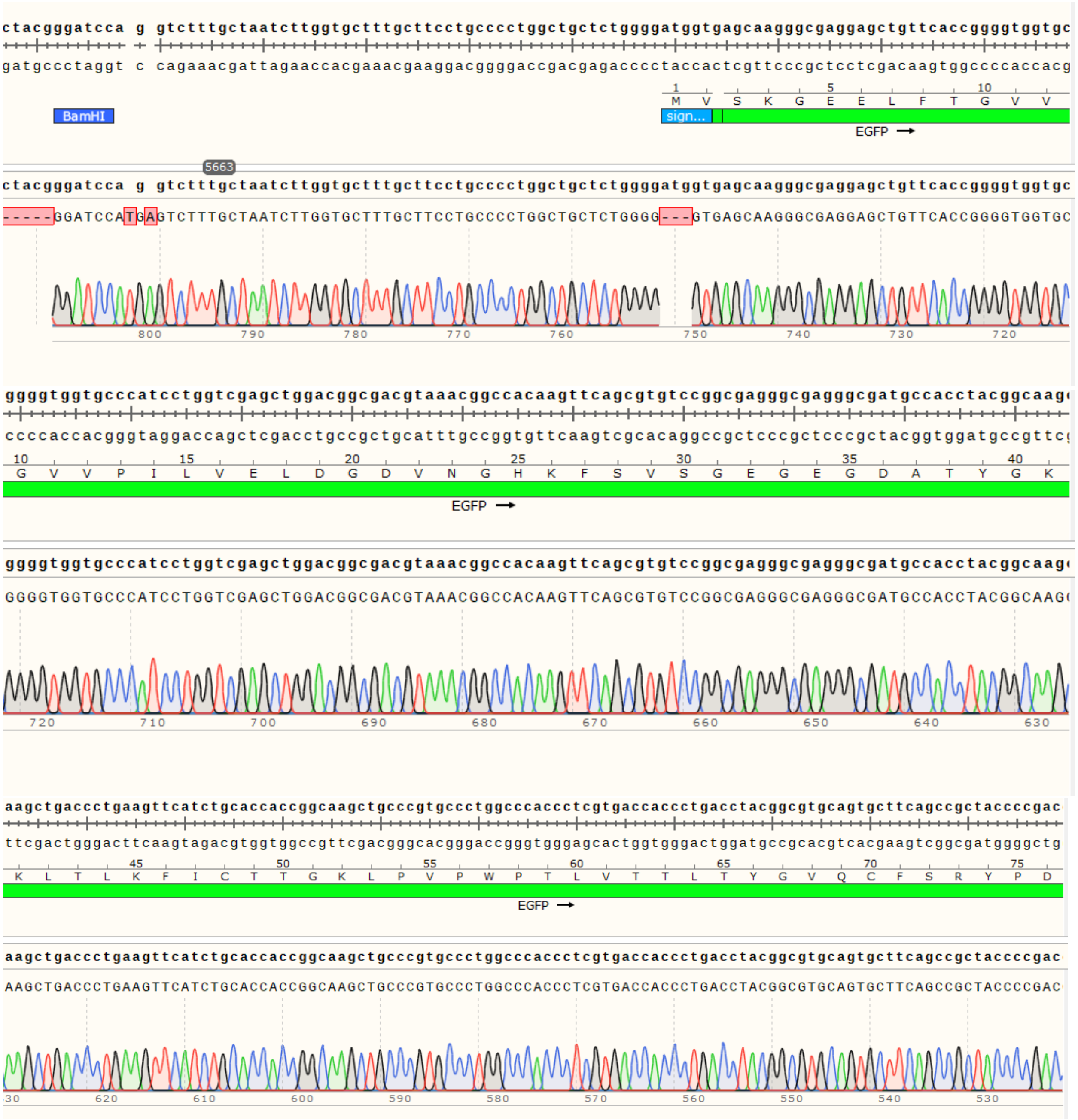

ctacgggatcca g gtctttgctaatcttggtgctttgcttcctgcccctggctgctctggggatggtgagcaagggcgaggagctgttcaccggggtggtgc
gatgccctaggt c cagaaacgattagaaccacgaaacgaaggacggggaccgacgagacccctaccactcgttcccgctcctcgacaagtggccccaccacg
M V S K G E E L F T G V V
BamHI
sign...
EGFP
5663
ctacgggatcca g gtctttgctaatcttggtgctttgcttcctgcccctggctgctctggggatggtgagcaagggcgaggagctgttcaccggggtggtgc
-----GGATCCATGAGTCTTTGCTAATCTTGGTGCTTTGCTTCCTGCCCCTGGCTGCTCTGGGG---GTGAGCAAGGGCGAGGAGCTGTTCACCGGGGTGGTGC
ggggtggtgcccatcctggtcgagctggacggcgacgtaaacggccacaagttcagcgtgtccggcgagggcgagggcgatgccacctacggcaag
ccccaccacgggtaggaccagctcgacctgccgctgcatttgccggtgttcaagtcgcacaggccgctcccgctcccgctacggtggatgccgttc
G V V P I L V E L D G D V N G H K F S V S G E G E G D A T Y G K
EGFP
ggggtggtgcccatcctggtcgagctggacggcgacgtaaacggccacaagttcagcgtgtccggcgagggcgagggcgatgccacctacggcaag
GGGGTGGTGCCCATCCTGGTCGAGCTGGACGGCGACGTAAACGGCCACAAGTTCAGCGTGTCCGGCGAGGGCGAGGGCGATGCCACCTACGGCAAG
aagctgaccctgaagttcatctgcaccaccggcaagctgcccgtgccctggcccaccctcgtgaccaccctgacctacggcgtgcagtgcttcagccgctaccccgac
ttcgactgggacttcaagtagacgtggtggccgttcgacgggcacgggaccgggtgggagcactggtgggactggatgccgcacgtcacgaagtcggcgatggggctg
K L T L K F I C T T G K L P V P W P T L V T T L T Y G V Q C F S R Y P D
EGFP
aagctgaccctgaagttcatctgcaccaccggcaagctgcccgtgccctggcccaccctcgtgaccaccctgacctacggcgtgcagtgcttcagccgctaccccgac
AAGCTGACCCTGAAGTTCATCTGCACCACCGGCAAGCTGCCCGTGCCCTGGCCCACCCTCGTGACCACCCTGACCTACGGCGTGCAGTGCTTCAGCCGCTACCCCGAC

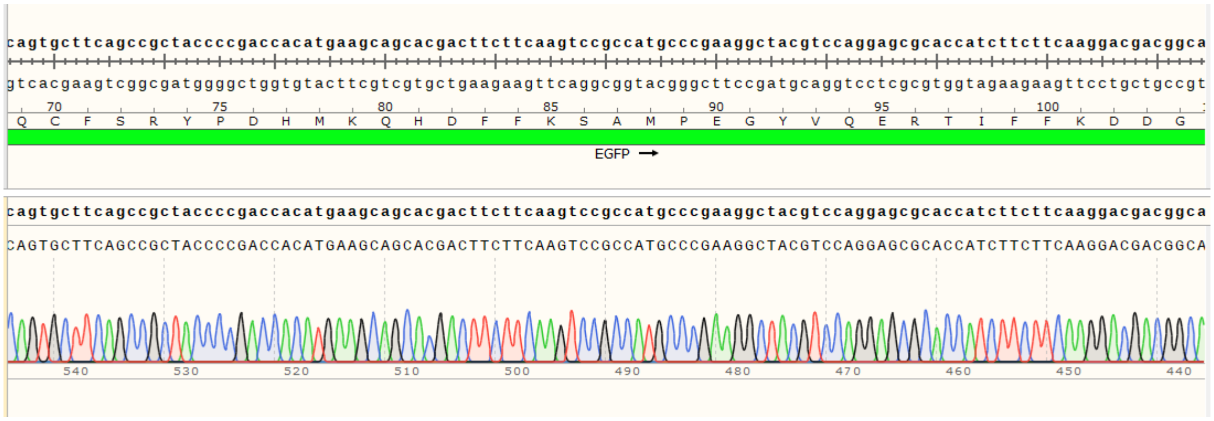
cagtgcttcagccgctaccccgaccacatgaagcagcacgacttcttcaagtccgccatgcccgaaggctacgtccaggagcgcaccatcttcttcaaggacgacggca
gtcacgaagtcggcgatggggctggtgtacttcgtcgtgctgaagaagttcaggcggtacgggcttccgatgcaggtcctcgcgtggtagaagaagttcctgctgccgt
70 75 80 85 90 95 100
Q C F S R Y P D H M K Q H D F F K S A M P E G Y V Q E R T I F F K D D G
EGFP
cagtgcttcagccgctaccccgaccacatgaagcagcacgacttcttcaagtccgccatgcccgaaggctacgtccaggagcgcaccatcttcttcaaggacgacggca
CAGTGCTTCAGCCGCTACCCCGACCACATGAAGCAGCACGACTTCTTCAAGTCCGCCATGCCCGAAGGCTACGTCCAGGAGCGCACCATCTTCTTCAAGGACGACGGCA
540 530 520 510 500 490 480 470 460 450 440

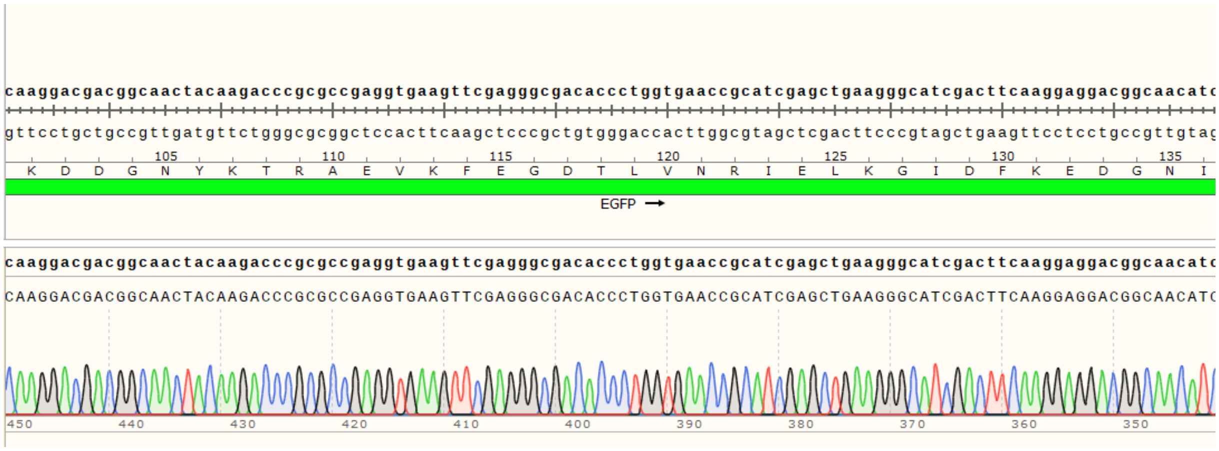
caaggacgacggcaactacaagacccgcgccgaggtgaagttcgagggcgacaccctggtgaaccgcatcgagctgaagggcatcgacttcaaggaggacggcaacatc
gttcctgctgccgttgatgttctgggcgcggctccacttcaagctcccgctgtgggaccacttggcgtagctcgacttcccgtagctgaagttcctcctgccgttgtag
105 110 115 120 125 130 135
K D D G N Y K T R A E V K F E G D T L V N R I E L K G I D F K E D G N I
EGFP
caaggacgacggcaactacaagacccgcgccgaggtgaagttcgagggcgacaccctggtgaaccgcatcgagctgaagggcatcgacttcaaggaggacggcaacatc
CAAGGACGACGGCAACTACAAGACCCGCGCCGAGGTGAAGTTCGAGGGCGACACCCTGGTGAACCGCATCGAGCTGAAGGGCATCGACTTCAAGGAGGACGGCAACATC
450 440 430 420 410 400 390 380 370 360 350

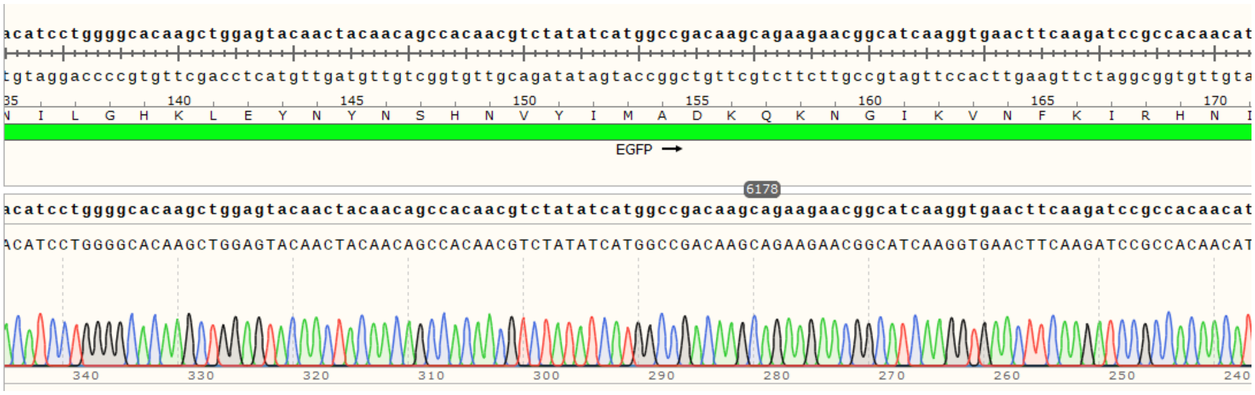
acatcctggggcacaagctggagtacaactacaacagccacaacgtctatatcatggccgacaagcagaagaacggcatcaaggtgaacttcaagatccgccacaacat
EGFP
6178
ACATCCTGGGGCACAAGCTGGAGTACAACTACAACAGCCACAACGTCTATATCATGGCCGACAAGCAGAAGAACGGCATCAAGGTGAACTTCAAGATCCGCCACAACAT

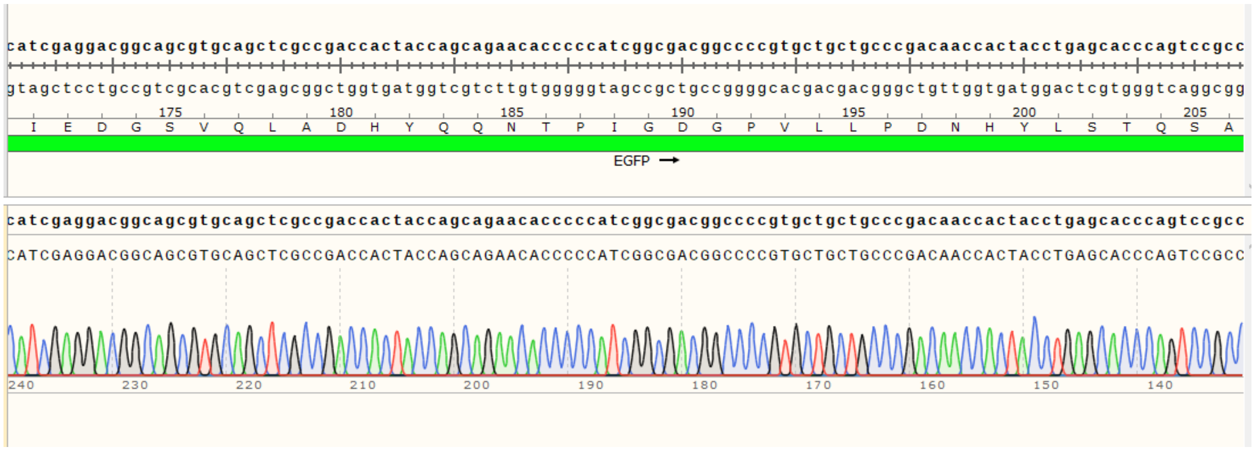
catcgaggacggcagcgtgcagctcgccgaccactaccagcagaacacccccatcggcgacggccccgtgctgctgcccgacaaccactacctgagcacccagtccgcc
EGFP
CATCGAGGACGGCAGCGTGCAGCTCGCCGACCACTACCAGCAGAACACCCCCATCGGCGACGGCCCCGTGCTGCTGCCCGACAACCACTACCTGAGCACCCAGTCCGCC

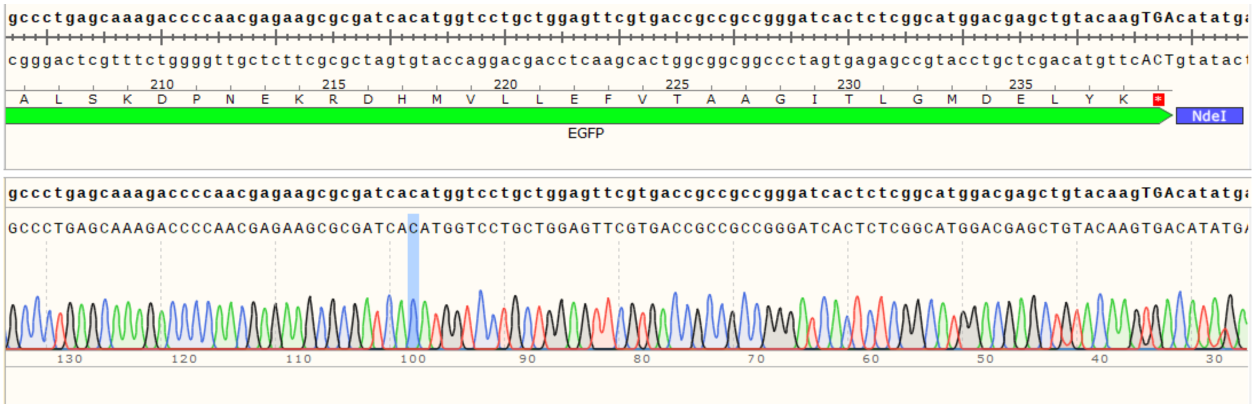
gccctgagcaaagaccccaacgagaagcgcgatcacatggtcctgctggagttcgtgaccgccgccgggatcactctcggcatggacgagctgtacaagTGAcatatg
EGFP
NdeI
GCCCTGAGCAAAGACCCCAACGAGAAGCGCGATCACATGGTCCTGCTGGAGTTCGTGACCGCCGCCGGGATCACTCTCGGCATGGACGAGCTGTACAAGTGACATATG
